# FOXM1 Is an Oncogenic Mediator in Ewing Sarcoma

**DOI:** 10.1371/journal.pone.0054556

**Published:** 2013-01-24

**Authors:** Laura Christensen, Jay Joo, Sean Lee, Daniel Wai, Timothy J. Triche, William A. May

**Affiliations:** 1 Division of Hematology-Oncology, Department of Pediatrics, Saban Research Institute, Childrens Hospital Los Angeles, Keck School of Medicine, University of Southern California, Los Angeles, California, United States of America; 2 Department of Pathology, Saban Research Institute, Childrens Hospital Los Angeles, Keck School of Medicine, University of Southern California, Los Angeles, California, United States of America; Virginia Commonwealth University, United States of America

## Abstract

Ewing Family Tumors (Ewing Sarcoma and peripheral Primitive Neuroectodermal Tumor) are common bone and soft tissue malignancies of childhood, adolescence and young adulthood. Chromosomal translocation in these tumors produces fusion oncogenes of the EWS/ETS class, with EWS/FLI1 being by far the most common. EWS/ETS chimera are the only well established driver mutations in these tumors and they function as aberrant transcription factors. Understanding the downstream genes whose expression is modified has been a central approach to the study of these tumors. FOXM1 is a proliferation associated transcription factor which has increasingly been found to play a role in the pathogenesis of a wide range of human cancers. Here we demonstrate that FOXM1 is expressed in Ewing primary tumors and cell lines. Reduction in FOXM1 expression in Ewing cell lines results in diminished potential for anchorage independent growth. FOXM1 expression is enhanced by EWS/FLI1, though, unlike other tumor systems, it is not driven by expression of the EWS/FLI1 target GLI1. Thiostrepton is a compound known to inhibit FOXM1 by direct binding. We show that Thiostrepton diminishes FOXM1 expression in Ewing cell lines and this reduction reduces cell viability through an apoptotic mechanism. FOXM1 is involved in Ewing tumor pathogenesis and may prove to be a useful therapeutic target in Ewing tumors.

## Introduction

Ewing Sarcoma is an aggressive malignancy of bone and soft tissue with a peak incidence in the adolescent/young adult years [1]. With intensive multimodal therapy, cure rates have modestly improved in recent years for those patients with localized disease. However, the outlook for those with metastatic or recurrent disease remains poor [2]. No definitive cell of origin has yet been described for this histologically primitive tumor which occurs in a diverse range of anatomic sites. However, a single molecular rearrangement unifies this tumor family. Virtually all Ewing tumors demonstrate a chromosomal rearrangement resulting in the fusion of the amino terminal domain of the EWS gene with the carboxy-terminal portion of an ETS transcription factor. The prototype rearrangement between chromosomes 11 and 22 produces an EWS/FLI1 fusion which is found in over 85% of Ewing tumors [3]. EWS/FLI1 and the other EWS/ETS chimeric proteins are thought to function as aberrant transcription factors [4]. These chimeric proteins have been shown to be critical to maintaining tumor phenotype in a variety of studies. Gene regulation imposed by EWS/FLI1 is felt to mediate important aspects of tumor phenotype. The details of gene deregulation by EWS/FLI1 have been extensively investigated [5].

FOXM1 is a member of the large evolutionarily conserved family of Forkhead box transcription factors [6]. FOXM1 is associated with proliferation and with cell cycle progression from G1-S and G2-M phases as well as mitotic chromosome stability [7]. Overexpression of FOXM1 has been shown to promote cell cycle progression [8]. Evidence suggests that FOXM1 is overexpressed in a variety of human cancers [9] including breast, gastric, and lung cancers [10] as well as glioma [11]. Of tumors common in pediatric oncology, FOXM1 has been implicated in the pathogenesis of neuroblastoma [12] and in medulloblastoma [13]. Its level of expression has been shown to inversely correlate with outcome in several tumor types [13], [14]. Multiple oncogenic functions have been attributed to FOXM1 in diverse tumor types, including proliferation, tumorigenicity, epithelial to mesenchymal transition, cell migration, and drug resistance [15]. The wide range of activities in multiple tumor systems makes FOXM1 a potentially inviting target for anticancer therapeutics. In basal cell carcinoma, FOXM1 expression has been shown to be dependent on GLI1 [16], the transcriptional effector of the Hedgehog-GLI pathway. Because of our interest in the role of the GLI1 transcription factors in Ewing tumors [17], [18], we became intrigued by a possible role for FOXM1 in Ewing tumor development.

In this paper, we show that FOXM1 is expressed at robust levels in a variety of Ewing tumor specimens and in Ewing cell lines. Furthermore, the level of expression of FOXM1 appears to be dependent on EWS/FLI1 in Ewing cell lines, though not on GLI1 expression in these same lines. Similar to a variety of other EWS/FLI1 driven targets, the expression of FOXM1 is important for Ewing cell line anchorage independent growth. Finally, to test the potential of drug targeting of FOXM1, we show that Thiostrepton, which directly targets FOXM1 expression and activity [19], also reduces Ewing cell line cell viability through an apoptotic mechanism. However, we do not find evidence that FOXM1 is directly targeted by EWS/FLI1. These findings suggest that FOXM1 may be an important mediator of EWS/FLI1 oncogenesis, though it may be indirectly targeted.

## Materials and Methods

### Microarray Data

Samples were processed at the Genomics Core at Children’s Hospital Los Angeles (CHLA) using the GeneChip Scanner 3000 7G System. RNA was extracted from samples and processed according to manufacturer’s instructions (Affymetrix, Inc, Santa Clara, CA). Samples were hybridized onto Human Exon 1.0 ST (HuEx) Arrays and processed as required.

Data analyses were performed at the Center for Personalized Medicine (CPM) at CHLA. HuEx CEL files were analyzed using Genomics Suite 6.6 (Partek, Inc, St. Louis, MO). Signal intensities for probe selection regions (PSRs) were quantile normalized by robust multichip averaging (RMA) using na32 annotations. Gene level analyses employed summarizing PSR expression.

### Real Time Quantitative PCR

RNA was extracted as previously described [17]. cDNA was synthesized using the Bio-Rad iScript cDNA Synthesis Kit. PCR was performed using iQ SYBR Green Supermix as previously described [17]. Specific primer sequences used are listed in Figure S4. All data presented was reproduced across at least three independent experiments.

### Western Blot

Western blot was performed as previously described [17] using antibody for FOXM1 (SC-502 from Santa Cruz Biotechnology), for Actin (SC-1616 from Santa Cruz Biotechnology), and for PARP (#9542 from Cell Signaling Technology).

### MTS Assay

Cells were cultured in variable amounts of drug for 72 hours and viable cell number was determined using an MTS assay (CellTiter 96® AQueous Non-Radioactive Cell Proliferation Assay, Promega) per manufacturer’s recommendations. All results represent at least three independent assays.

### Drug Treatment

Siomycin was obtained from the NCI DTP program (http://dtp.nci.nih.gov). Thiostrepton was obtained from Millipore/EMD. Both drugs were dissolved in DMSO (Sigma) and were used at the indicated concentrations. All results represent at least three independent assays.

### Plasmid Constructs Used

EWS/FLI1 expression constructs were Type 4 EWS/FLI1 cDNA cloned into either pLXIN or pLXIH. The HA tagged human GLI1 retroviral expression construct was the cDNA from pCS2HA3huGli1dlta2EcoR1 plasmid [20] kindly provided by Dr. A. E. Oro. The ORF was amplified by PCR and this was cloned with the InFusion Cloning System (Clontech) into the BamH1 site of pLXIN (Clontech).

### Retroviral shRNA Experiments

These were conducted as previously described [18]. The sequence of shRNA used is included in Figure S4.

### Reporter Gene Experiments

A primers set was used to amplify the genomic region from +21 nucleotides and -1300 relative to the transcription start site. FOXM1-1322s (CAC ACC ACA CTC CAT TCA GG) and FOXM1-1322as (CAG TTT GTT CCG CTG TTT GA). This amplicon was cloned into the Xho1 site of pLightSwitch_Prom (SwitchGear Genomics) using InFusion Cloning System (Clontech). The insert was sequenced for confirmation.

NIH 3T3 cells were plated to 70–90% confluence in 96 well plates and transfected with a mixture of 0.2 mcg of total DNA and 1.5 mcl of Lipofectamine 2000 (Invitrogen) per well along with 1/100 of CMV Luciferase control [21]. 0.025 mcg of the DNA was reporter plasmid. Increasing amounts of EWS/FLI1 driver were used starting at 0.02 mcg per tranfected well. Cells were cultured for 72 hours and were assayed with the Dual-Glo Luciferase Assay System (Promega) per the manufacturer’s protocol. Reported results represent at least three independent assays.

### Cells, Cell Culture, and Materials

All tumor cell lines were grown in RPMI supplemented with 2 mM glutamine and 10% Fetal Bovine Serum at 37 degrees C and in 5% CO_2_. Parental NIH3T3 cells and those cell lines derived from them were grown in DMEM in 5% bovine calf serum. All human cell lines were obtain from Dr. C. Patrick Reynolds via the Ewing Preclinical Testing Lab (www.EFTlab.org).

### Statistics

All statistical comparisons are made by an unpaired Student’s t-test.

## Results

### Ewing Tumors Express FOXM1

**Figure 1:**
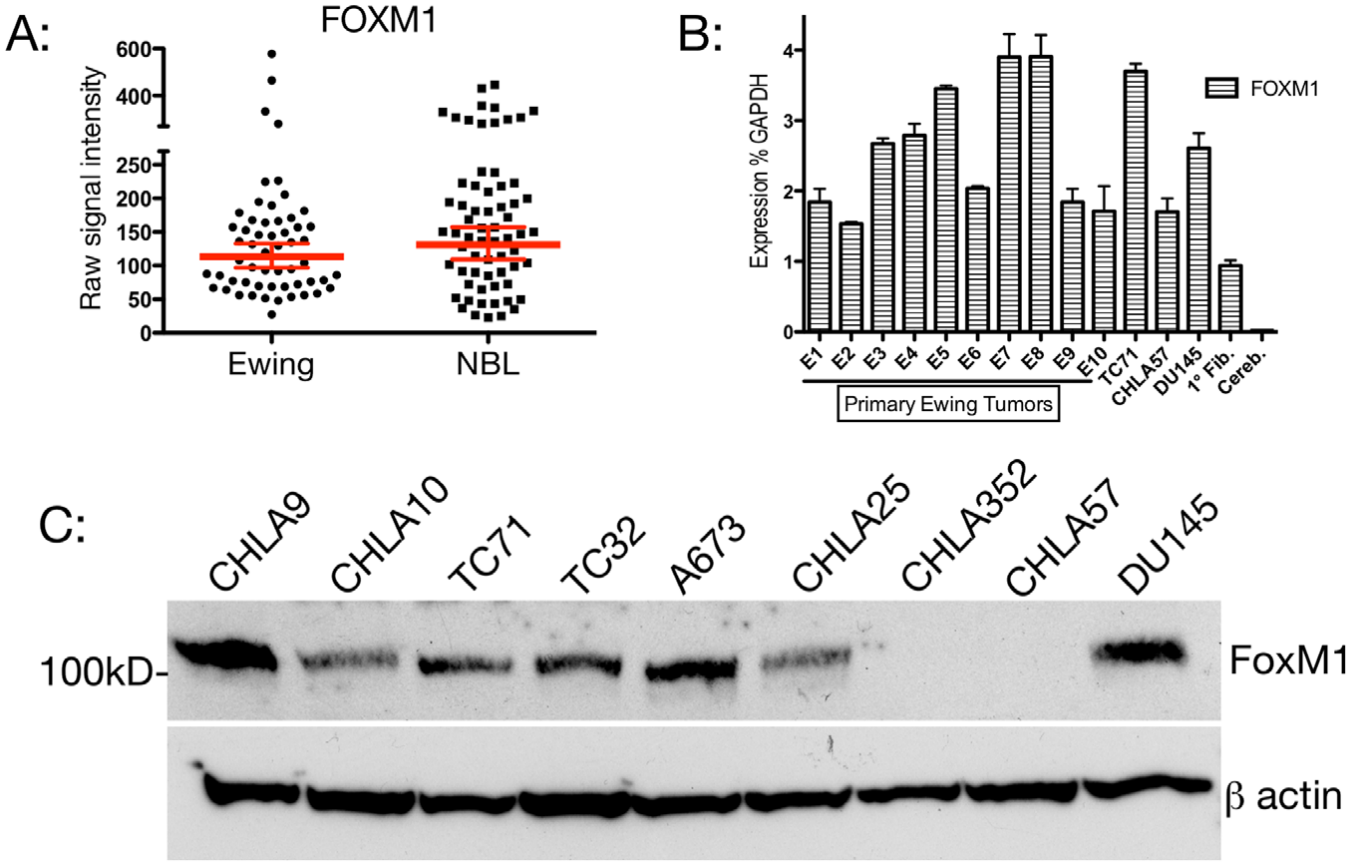
FOXM1 expression in Ewing Sarcoma. A: Microarray data from Affymetrix Human Exon 1.0 ST (HuEx) Arrays reveal FOXM1 expression levels which are comparable to Neuroblastoma (NBL) and not statistically different (p = 0.12). B: Real time quantitative PCR for FOXM1 (all splice forms) confirms significant levels of transcript in primary Ewing Sarcoma tumor specimens and the Ewing cell line TC71, compared to CHLA57, a Schwannoma cell line, DU145, a Prostate Carcinoma cell line, cultured primary fibroblasts (Fibr.) and mature cerebellar tissue (Cereb.). C: Western blot for FOXM1 detects FOXM1 protein in 6/7 Ewing cell lines tested and in the Prostate cell line DU145.

FOXM1 expression has been found to be elevated in a wide variety of human malignancies. To evaluate a possible role of FOXM1 in Ewing tumor biology, we evaluated microarray data from 56 patients with localized Ewing Sarcoma. Neuroblastoma is a biologically distinct Pediatric Solid Tumor in which a biologic role for FOXM1 has been recently described [12]. We therefore compared microarray expression levels from our Ewing primary tumors with a set of Neuroblastoma specimens. We found no significant difference in microarray expression levels between the two patient groups (Figure 1A). To confirm these data, we assessed the level of FOXM1 transcript by qPCR in both primary Ewing specimens and in several Ewing cell lines. Figure 1B demonstrates robust transcript levels by this method, confirming our microarray findings. Finally, Figure 1C shows that these transcript levels correlate with FOXM1 protein which is easily detectable on Western Blot. These data indicate that FOXM1 expression in Ewing sarcoma is comparable to that observed in other tumor systems. Given the role established for FOXM1 in other tumor systems, we undertook to determine whether it plays a similar role in Ewing tumors.

### shRNA to FOXM1 Impairs Ewing Cell Line Growth

**Figure 2:**
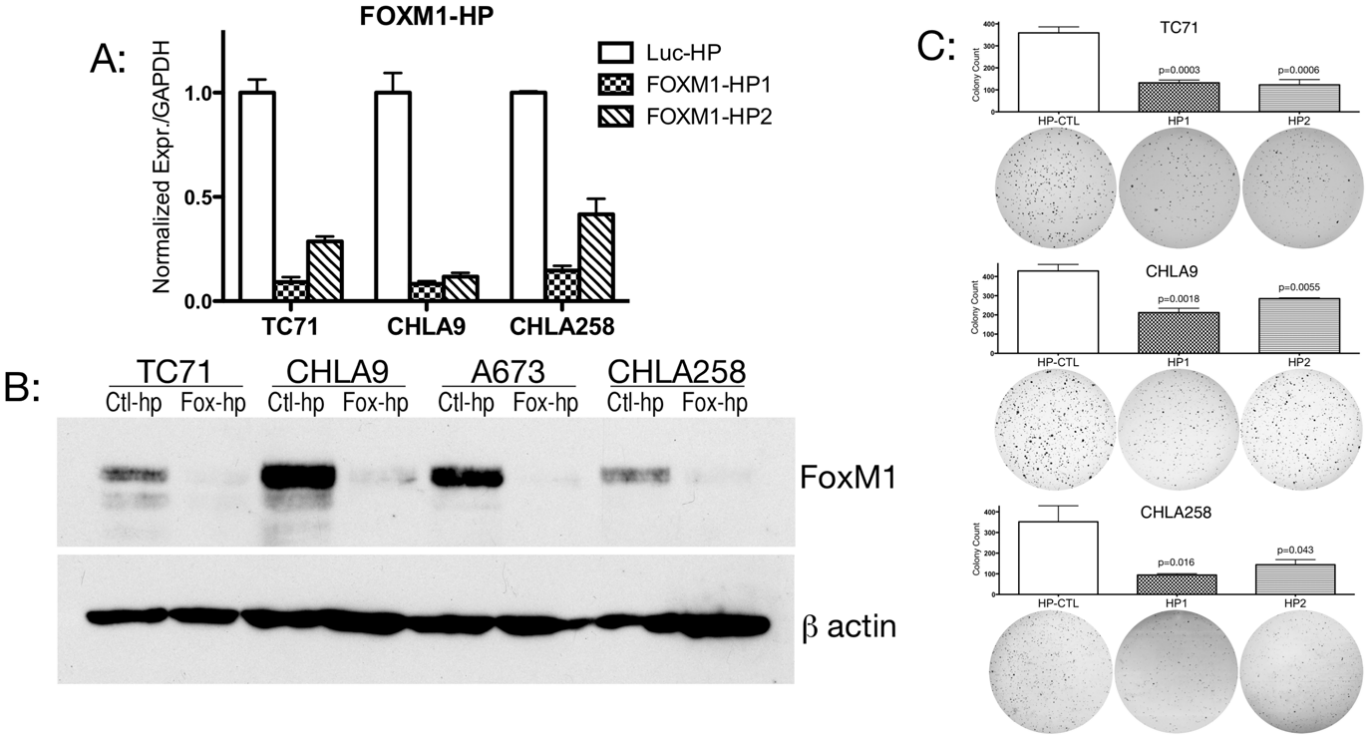
FOXM1 knockdown reduces anchorage independent growth in Ewing cell lines. A: Three Ewing cell lines were transduced with either of two FOXM1 lentiviral shRNA or a nontargeting Luciferase control and polyclonal lines were selected with Puromycin. Real time quantitative PCR for FOXM1 shows significant reduction in FOXM1 transcript. All differences significant to p<$>\scale 60%\raster="rg1"<$>0.05 by Student’s t-test. B: Cell lines transduced with FOXM1-HP1 confirm a significant reduction in FOXM1 protein. C: Three Ewing cell lines with FOXM1 shRNA are plated in soft agar and compared to non-targeting control. All three demonstrate reduction in colony formation with either FOXM1 shRNA.

To assess the potential role of FOXM1 expression in driving abnormal cell growth in Ewing cells, we constructed lentiviral shRNA to FOXM1 using target sequences shown effective in other systems [22], [23]. Ewing cells lines were transduced with lentiviral particles and polyclonal cell lines were rapidly selected with Puromycin. Figure 2A shows the reduction in FOXM1 transcript in several Ewing cell lines transduced with either of these shRNA. Furthermore, this reduction in transcript produces a significant reduction in FOXM1 protein levels (Figure 2B). We next sought to measure effects on oncogenic proliferation in Ewing cell lines. Targeted knockdown of EWS/FLI1 itself and multiple downstream targets of EWS/FLI1 has been extensively studied in vitro by anchorage independent growth assays [4], [21], [24], [25]. We therefore tested the effect of this shRNA mediated reduction in FOXM1 expression on the anchorage independent growth of several Ewing lines. We seeded cells in single cell suspension and incubated for 2–3 weeks. Figure 2C shows that the reduction of FOXM1 expression impairs the ability of these Ewing cell lines to grow in an anchorage independent fashion. This indicates that FOXM1 expression is important to Ewing tumor cell proliferation similar to EWS/FLI1 and to several previously described EWS/FLI1 targets.

### EWS/FLI1 Upregulates FOXM1 in Ewing Cells

**Figure 3:**
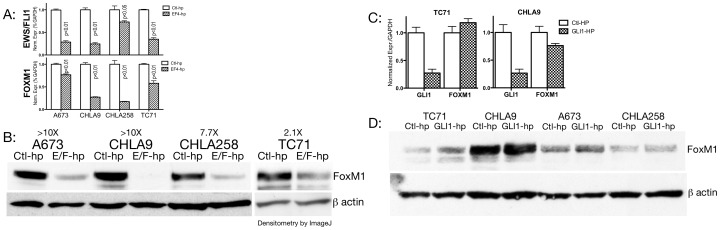
FOXM1 expression in Ewing cell lines is EWS/FLI1 dependent but not GLI1 dependent. A: EWS/FLI1 lentiviral shRNA or a non-targeting control was used to derive polyclonal populations in four Ewing cell lines. By real time quantitative PCR, all demonstrate reduced EWS/FLI1 transcript and reduced FOXM1 transcript. All differences significant to p<$>\scale 60%\raster="rg1"<$>0.05 by Student’s t-test as shown. B: Western blot for FOXM1 on the same cell lines demonstrates consistent reduction in FOXM1 protein. Fold change in band intensity is quantitated with ImageJ. C: Ewing cell lines transduced with GLI1 shRNA demonstrate reduction in the GLI1 transcript (p<$>\scale 60%\raster="rg1"<$>0.05). However, no significant change in FOXM1 transcript is detected. D: Western blot of Ewing cell lines treated with GLI1 shRNA also reveals no reduction in FOXM1 protein.

Since EWS/FLI1 is the best established driver mutation in Ewing tumors, we sought to determine whether EWS/FLI1 can drive the expression of FOXM1. We employed previously characterized lentiviral shRNA to EWS/FLI1 to derive polyclonal cell lines for both EWS/FLI1 specific shRNA and for a non-targeting control. Figure 3A shows that in four tested Ewing lines, shRNA mediated knockdown of EWS/FLI1 expression results in a reduction of FOXM1 transcript. Furthermore, Figure 3B demonstrates that EWS/FLI1 shRNA knockdown also results in decreased levels of FOXM1 protein across four cell lines. These data indicate that EWS/FLI1 modulates FOXM1 expression, either by direct or indirect mechanisms.

We have previously demonstrated the importance of GLI1 as downstream target of EWS/FLI1 [17] and that some targets of EWS/FLI1 appear to be indirectly regulated through GLI1 [18]. Subsequently, it has been demonstrated that GLI1 is a direct transcriptional target of EWS/FLI1 [26]. Since GLI1 has been shown to drive expression of FOXM1 in basal cell carcinoma [16], there is an appealing logic to the hypothesis that EWS/FLI1 indirectly targets FOXM1 through deregulation of GLI1. We therefore sought to determine if GLI1 played a role in Ewing tumors much like the role it plays in basal cell carcinoma. However, Figure 3C shows that, even with effective shRNA mediated reduction of GLI1 in Ewing cell lines, there is no effect on FOXM1 expression. Furthermore, overexpression of GLI1 in Ewing cell lines also fails to produce any change in FOXM1 expression (Figure S1). We conclude that while FOXM1 is activated by EWS/FLI1, it is not in the subset of EWS/FLI1 targets that are deregulated via GLI1 deregulation.

Finally, in an attempt to determine if FOXM1 might be directly targeted by EWS/FLI1, we constructed a reporter placing the FOXM1 promoter up to nucleotide -1300 upstream of the transcription start site in the pLightSwitch_Prom luciferase reporter vector. This construct was transfected into NIH3T3 cells, a well established cell line for assessing EWS/FLI1 biology [4], along with an expression construct for EWS/FLI1 or a vector control. In multiple experiments, we were unable to demonstrate any significant (i.e. greater than 1.2–1.4x) increase in the luciferase reporter gene (data not shown). Further experiments with a commercially available FOXM1 reporter (from SwitchGear Genomics) were also unsuccessful. We therefore find no evidence that FOXM1 is directly targeted by EWS/FLI1 within the scope of this analysis.

### Thiostrepton Reduces FOXM1 Expression in Ewing Cell Lines, Resulting in Decreased Cell Viability via an Apoptotic Mechanism

The widespread evidence of the importance of FOXM1 in various tumor types has led to speculation that it might prove to be a useful drug target in a wide spectrum of diseases. Recent evidence has pointed to FOXM1 inhibition resulting from treatment with Thiazole antibiotics. Siomycin A has been shown to decrease FOXM1 expression with resulting apoptotic cell death in several tumor systems [27]–[29]. The mechanism may be related to the activity of the compound as a proteosomal inhibitor [28]. More recently, the naturally occurring antibiotic Thiostrepton has been shown to have similar effects [29], [30]. In addition to functioning as a proteosomal inhibitor [31], Thiostrepton has been shown to physically interact with FOXM1 and to consequently inhibit FOXM1 binding to target promoters [19]. We therefore decided to measure the effect of these compounds against Ewing cell lines.

**Figure 4:**
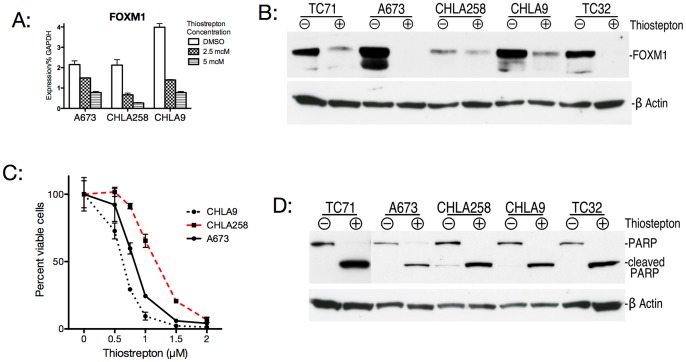
Thiostrepton reduces FOXM1 expression in Ewing cell lines, producing diminished cell viability by an apoptotic mechanism. A: Ewing cell lines were treated for 16 hours with Thiostrepton at increasing concentrations. Real time quantitative PCR shows a reduction of FOXM1 transcript. B: Ewing cell lines treated with 5 mcM Thiostrepton for 36–72 hours show decreased FOXM1 protein on western blot. C: Treatment of Ewing cell lines with increasing quantities of Thiostrepton for 72 hours results in reduced numbers of viable cells compared to diluent controls. D: Ewing cell lines treated as in panel B show cleavage of PARP, an indicator of apoptosis.

Figure 4A shows the effects of treating several Ewing cell lines with Thiostrepton. Consistent with findings in other tumor systems, there is significant reduction in FOXM1 transcript with overnight treatment in the low micromolar range. This reduction in transcript is reflected in a reduction in FOXM1 protein with slightly extended incubation (36 to 72 hours). Since reduced cell viability has been shown to be a consequence of Thiostrepton treatment in other malignancies, we used an MTS assay to measure cell viability at increasing concentrations of Thiostrepton for 72 hours incubation. We found significant drops in cell viability in the low micromolar range. In other systems, this reduction in viability was found to result from an apoptotic mechanism. We therefore assessed protein extracts from treated cells and found that cleaved PARP, a marker of apoptotic cell death, was increased in treated Ewing cell lines. We found very similar results with Siomycin A (Figure S2). This apoptotic effect of these compound also correlates with an increase in cleaved PARP seen in Ewing cell lines treated with lentiviral shRNA (Figure S3). This indicates that at least some of the apoptosis due to these compounds is due to a reduction in FOXM1 levels.

These peptide antibiotics show in vitro activity in Ewing cell lines similar to that seen in other tumor systems. Along with our shRNA data, this suggests FOXM1 may prove a useful therapeutic target in Ewing tumors.

## Discussion

While facilitating and cooperative mutations may yet be identified, for the present time the operative functional model for Ewing tumor biology is for a single class of driver mutation, EWS/ETS fusion oncogenes, to mediate critical aspects of tumor biology. A preponderance of evidence suggests the EWS/ETS oncogenes primarily function via aberrant transcriptional targeting of downstream genes and pathways. Some examples of newer targeted therapies can bring dramatic responses which can prove fleeting as resistance rapidly emerges [32]. Therefore, even if efforts to directly target EWS/FLI1 [33] eventually bear clinical fruit, it is unlikely that any single targeted therapy would be curative. Therefore, biologically relevant secondary targets need to be identified. [34].

The data we present suggests that FOXM1 may be such a target. Its expression in Ewing tumors is comparable to Neuroblastoma, another childhood solid tumor in which FOXM1 has been studied. RNAi mediated inhibition of FOXM1 inhibits Ewing cell line growth. EWS/FLI1 upregulates FOXM1 though, interestingly, it is not targeted via GLI1. While it is possible [12] that EWS/FLI1 is directly mediated through some distant enhancer element, the core promoter region plus further upstream elements of the FOXM1 promoter region does not give evidence of direct transcriptional upregulation in an NIH3T3 cell model. Since FOXM1 is a cell cycle mediator, it is possible that EWS/FLI1 may induce FOXM1 as an indirect consequence of other targeted pathways which drive proliferation and entry into the cell cycle. Indeed many modulators of FOXM1 have been described, including E2F [35], ERK [36], HIF-1 [37], and AKT [34], so the potential for indirect upregulation is significant. In any case, the biological and potential therapeutic potential of FOXM1 is demonstrated in principal by the action of the thiazole antibiotic Thiostrepton. This agent, which may in part act by directly binding FOXM1, appears to reduce the expression of FOXM1 and to reduce the number of viable cells in culture via an apoptotic mechanism. These effects are similar to those seen in other tumor systems. So FOXM1 appears to be of biologic significance in Ewing tumors in vitro but also can, in principle, be targeted via small molecule inhibition.

Our finding of FOXM1 inhibition by Thiostrepton in Ewing cells suggests that compounds of this class may be of potential utility in the treatment of Ewing tumors. The mechanism by which Thiostrepton inhibits FOXM1 has been proposed to be via direct binding of FOXM1 [19] and also via its activity as a protease inhibitor [28]. Of note, another inhibitor, Bortezomib, has been shown to act on FOXM1 similarly to Thiostrepton and Siomycin [28], though there is no evidence for direct binding of FOXM1. Bortezomib has shown activity in preclinical in vitro Ewing models [38]. However, little activity was noted in testing against xenograft models of Ewing sarcoma [39]. Also, results from a Phase 2 trial of Bortezomib against a variety of sarcomas have been unencouraging [40]. Nevertheless, Thiostrepton, in its ability to bind FOXM1 directly, may possess activities which other protease inhibitors lack.

The intriguing aspect of FOXM1 is that it is so widely overexpressed and means to target it appear so consistently effective in preclinical models. Given the rarity of all Pediatric cancers, bringing to market compounds whose efficacy is limited to a specific class of pediatric tumors may not be a viable strategy on economic grounds. Therefore therapeutic strategies aimed at targets and pathways which can also apply to more prevalent human tumors appear much more likely to successfully survive the drug development process. Our data demonstrate that targeting FOXM1 has the potential to therapeutically bridge the worlds of pediatric and adult oncology.

## Supporting Information

Figure S1 **Overexpression of GLI1 does not increase FOXM1.** Three Ewing cell lines were transduced with retroviral vector control or with an HA tagged form of GLI1. Polyclonal lines were selected with Puromycin. Protein lysates were assessed on Western Blot. All lines showed GLI1 overexpression but none showed any increase in endogenous FOXM1.
(PDF)

Figure S2 **Siomycin in Ewing Cell lines.** A: qPCR for FoxM1 in two Ewing cell lines treated with 2,5 mcM of Siomycin shows diminished FOXM1 transcript. B: Ewing cell lines treated with increasing concentrations of Siomycin show greatly diminished FOXM1 protein. C: Siomycin treatment of Ewing cell lines decreases cell viability measured by an MTS assay. D: Ewing cell lines treated with Siomycin demonstrate increased cleavage of PARP, a marker of apoptosis.
(PDF)

Figure S3 **shRNA Mediated Reduction in FOXM1 also Enhances Apoptosis in Ewing Cell Lines.** Cell lines were transduced with Lentiviral shRNA to FOXM1 or with a non-targeting control (minus sign). Western blot shows that cleaved PARP is enhanced this alternate method of FOXM1 reduction.
(PDF)

Figure S4 **Sequence of oligonucleotides employed.**
(PDF)
