# Supplementary material for: FOXM1 Is an Oncogenic Mediator in Ewing Sarcoma
Source: PLoS One. 2013 Jan 24;8(1):e54556. doi: 10.1371/journal.pone.0054556 (PMC3554707; doi:10.1371/journal.pone.0054556)

Figure S1: Overexpression of GLI1 does not increase FOXM1
Three Ewing cell lines were transduced with retroviral vector control or with an HA tagged form of GLI1. Polyclonal lines were selected with Puromycin. Protein lysates were assessed on Western Blot. All lines showed GLI1 overexpression but none showed any increase in endogenous FOXM1.

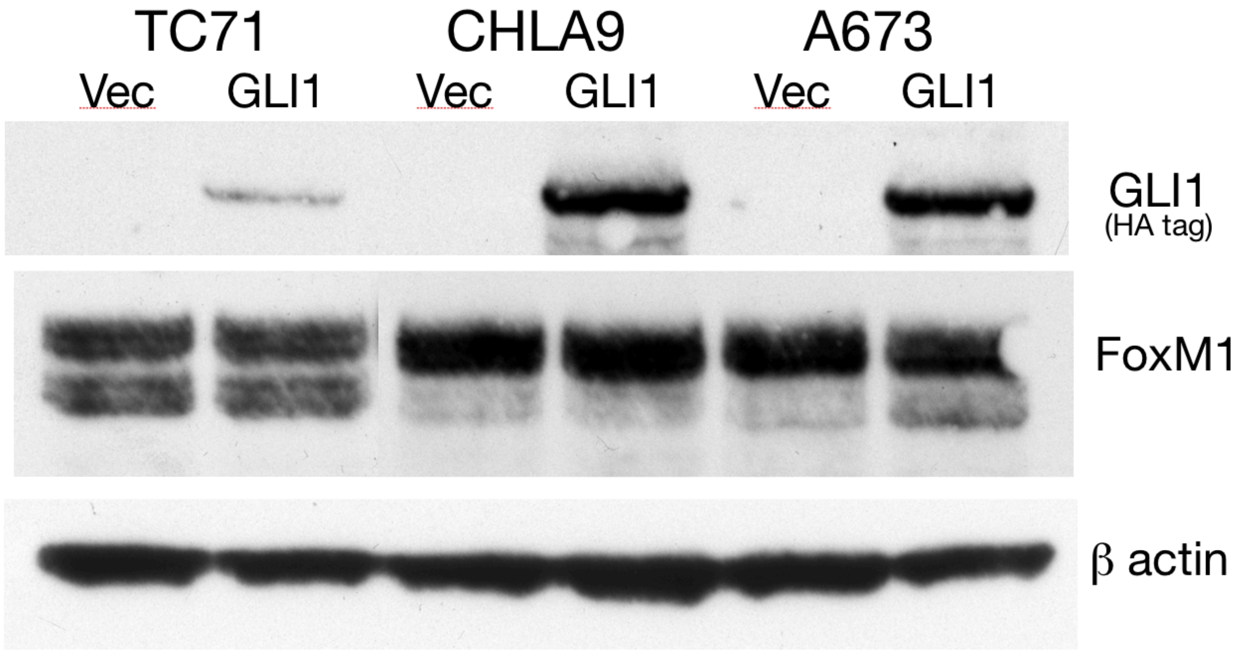

Supplement: Figure S1 — Overexpression of GLI1 does not increase FOXM1. Three Ewing cell lines were transduced with retroviral vector control or with an HA tagged form of GLI1. Polyclonal lines were selected with Puromycin. Protein lysates were assessed on Western Blot. All lines showed GLI1 overexpression but none showed any increase in endogenous FOXM1. (PDF) [file pone.0054556.s001.pdf]
