# Supplementary material for: FOXM1 Is an Oncogenic Mediator in Ewing Sarcoma
Source: PLoS One. 2013 Jan 24;8(1):e54556. doi: 10.1371/journal.pone.0054556 (PMC3554707; doi:10.1371/journal.pone.0054556)
Supplement: Figure S2 — Siomycin in Ewing Cell lines. A: qPCR for FoxM1 in two Ewing cell lines treated with 2,5 mcM of Siomycin shows diminished FOXM1 transcript. B: Ewing cell lines treated with increasing concentrations of Siomycin show greatly diminished FOXM1 protein. C: Siomycin treatment of Ewing cell lines decreases cell viability measured by an MTS assay. D: Ewing cell lines treated with Siomycin demonstrate increased cleavage of PARP, a marker of apoptosis. (PDF) [file pone.0054556.s002.pdf]

Figure S2: Siomycin in Ewing Cell lines.
A: qPCR for FoxM1 in two Ewing cell lines treated with 2,5 mcM of Siomycin shows diminished FOXM1 transcript. B: Ewing cell lines treated with increasing concentrations of Siomycin show greatly diminished FOXM1 protein. C: Siomycin treatment of Ewing cell lines decreases cell viability measured by an MTS assay. D: Ewing cell lines treated with Siomycin demonstrate increased cleavage of PARP, a marker of apoptosis.

A:

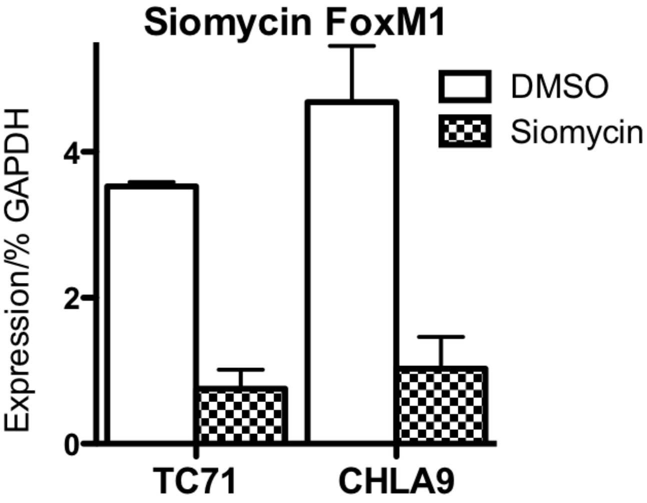

B:

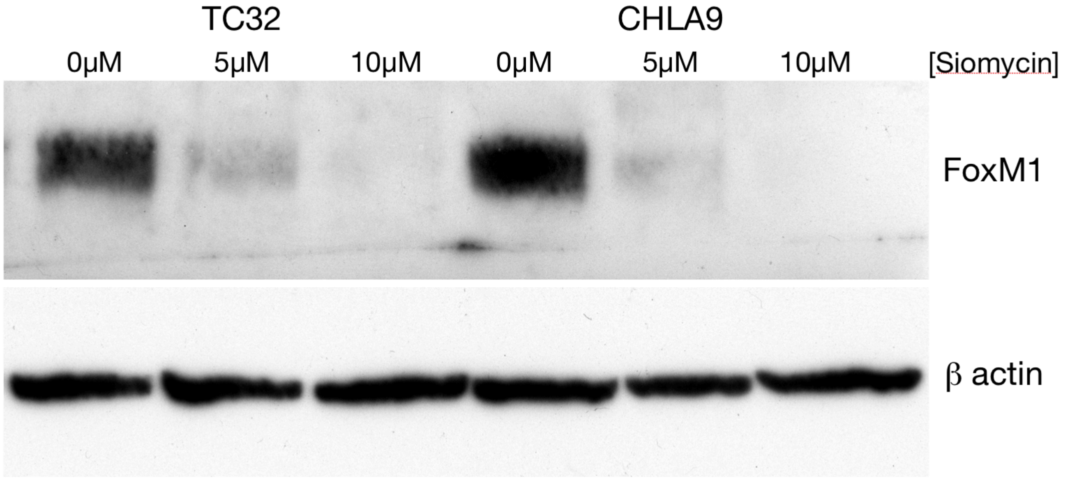

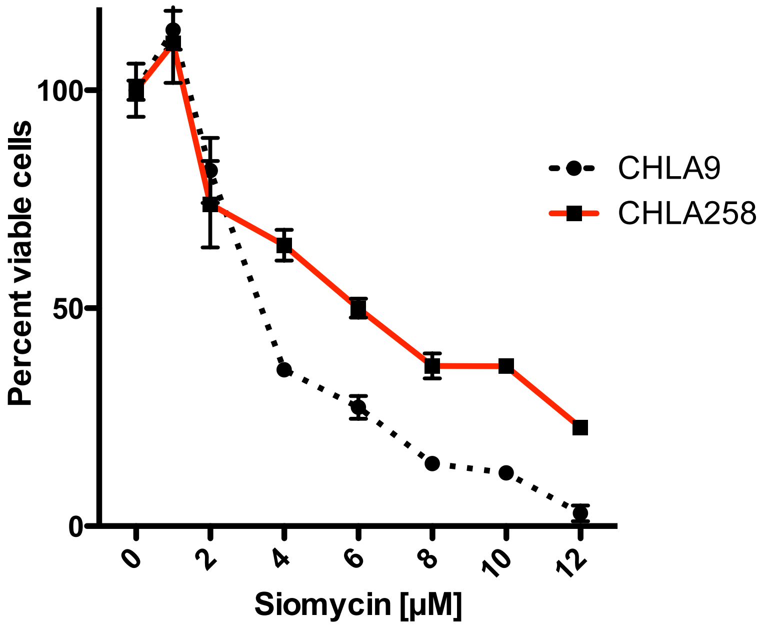
Percent viable cells
100
50
0
0
2
4
6
8
10
12
Siomycin [μM]
CHLA9
CHLA258

C:

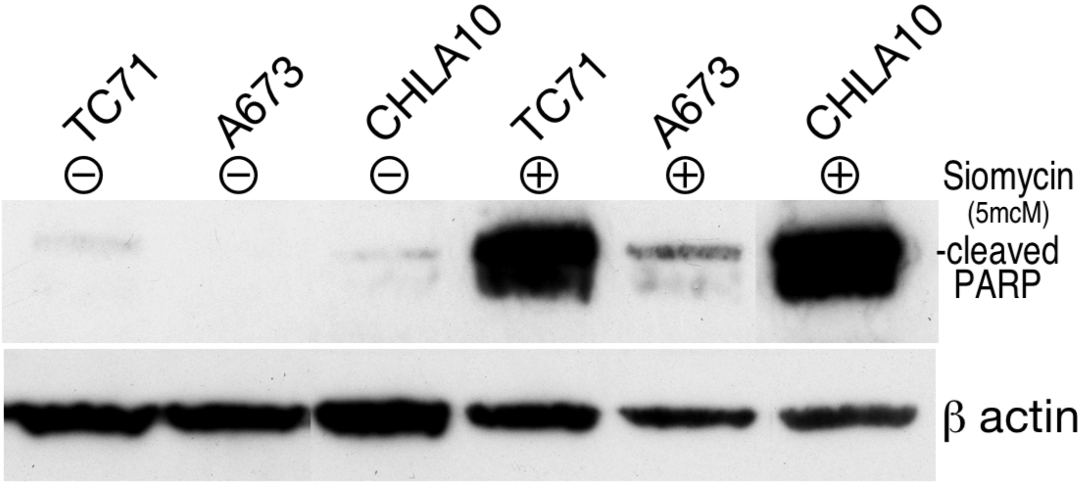
TC71
A673
CHLA10
TC71
A673
CHLA10
Siomycin
(5mcM)
-cleaved
PARP
β actin

D:
