# Supplementary material for: FOXM1 Is an Oncogenic Mediator in Ewing Sarcoma
Source: PLoS One. 2013 Jan 24;8(1):e54556. doi: 10.1371/journal.pone.0054556 (PMC3554707; doi:10.1371/journal.pone.0054556)

Figure S3: shRNA Mediated Reduction in FOXM1 also Enhances Apoptosis in Ewing Cell Lines. Cell lines were transduced with Lentiviral shRNA to FOXM1 or with a non-targeting control (minus sign). Western blot shows that cleaved PARP is enhanced this alternate method of FOXM1 reduction.

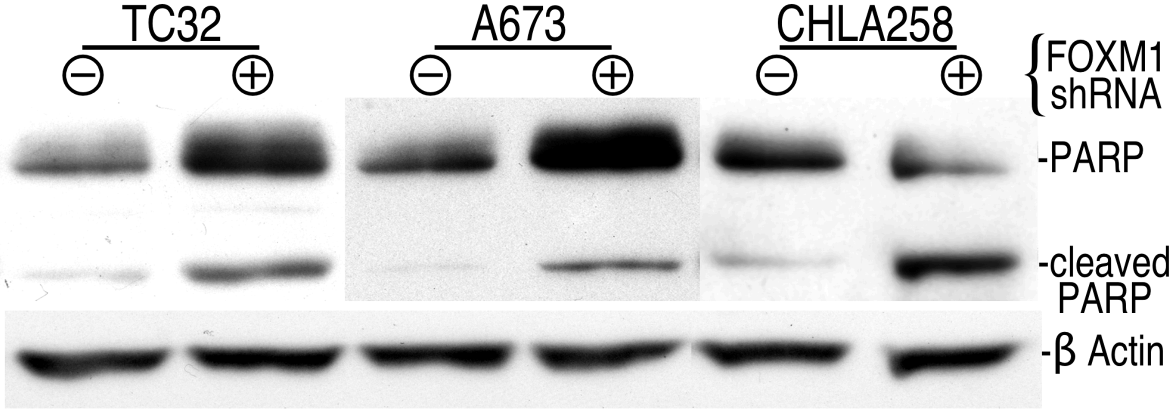

Supplement: Figure S3 — shRNA Mediated Reduction in FOXM1 also Enhances Apoptosis in Ewing Cell Lines. Cell lines were transduced with Lentiviral shRNA to FOXM1 or with a non-targeting control (minus sign). Western blot shows that cleaved PARP is enhanced this alternate method of FOXM1 reduction. (PDF) [file pone.0054556.s003.pdf]
