# Supplementary material for: FOXM1 Is an Oncogenic Mediator in Ewing Sarcoma
Source: PLoS One. 2013 Jan 24;8(1):e54556. doi: 10.1371/journal.pone.0054556 (PMC3554707; doi:10.1371/journal.pone.0054556)
Supplement: Figure S4 — Sequence of oligonucleotides employed. (PDF) [file pone.0054556.s004.pdf]

Figure S4: Sequence of oligonucleotides employed

qPCR Primer sequences: (5’-sense-3’//5’-antisense-3’):

FoxM1.4 GCAGGCTGCACTATCAACAA//TCGAAGGCTCCTCAACCTTA
Human GLI1-ALT: CTTGTGGTCCCCATGACTCT//GATTCAGGCTCACGCTTCTC
EWS/FLI1: CGACTAGTTATGATCAGAGCAGT//CCGTTGCTCTGTATTCTTACTGA

shRNA Oligo Sequences:

EWS/FLI1 Hairpin (EF-4)

FW: GATCCGGACGCCAAGGGCATTGCAG<u>TTCAAGAGA</u>CTGCAATGCCCTTGGCGTCTTTTTG
RV: AATTCAAAAAGACGCCAAGGGCATTGCAGTCTCTTGAACTGCAATGCCCTTGGCGTCCG

GLI1 Hairpin (GLI2757)

FW: GATCCGGGCTCAGCTTGTGTGTAATTTCAAGAGAATTACACACAAGCTGAGCCTTTTTG
RV: AATTCAAAAAGGCTCAGCTTGTGTGTAATTCTCTTGAAATTACACACAAGCTGAGCCCG

FoxM1-HP1

5'-GATCCGCCTTTCCCTGCACGACATGTTCAAGAGACATGTCGTGCAGGGAAAGGTTTTTTGGAAG-3'
5'-AATTCTTCCAAAAAACCTTTCCCTGCACGACATGTCTCTTGAACATGTCGTGCAGGGAAAGGCG-3'
(derived from Cancer Res 2005; 65: (12). June 15, 2005)

FoxM1-HP2

5'-GATCCGCTCTTCTCCCTCAGATATATTCAAGAGATATATCTGAGGGAGAAGAGTTTTTTGGAAG-3'
5'-AATTCTTCCAAAAAACTCTTCTCCCTCAGATATATCTCTTGAATATATCTGAGGGAGAAGAGCG-3'
(derived from Cancer Res 2006; 66: (7). April 1, 2006)
